# Effectiveness of additional self-care acupressure for women with menstrual pain compared to usual care alone: using stakeholder engagement to design a pragmatic randomized trial and study protocol

**DOI:** 10.1186/1745-6215-14-99

**Published:** 2013-04-11

**Authors:** Susanne Blödt, Lena Schützler, Wenjing Huang, Daniel Pach, Benno Brinkhaus, Josef Hummelsberger, Barbara Kirschbaum, Kirsten Kuhlmann, Lixing Lao, Fanrong Liang, Anna Mietzner, Nadine Mittring, Sabine Müller, Anna Paul, Carolina Pimpao-Niederle, Stephanie Roll, Huangan Wu, Jiang Zhu, Claudia M Witt

**Affiliations:** 1 Institute for Social Medicine, Epidemiology and Health Economics, Charité Universitätsmedizin - Berlin, Luisenstraße 57, Berlin, D- 10117, Germany; 2 Chengdu University of Traditional Chinese Medicine, Chengdu, China; 3 International Society for Chinese Medicine (Societas Medicinae Sinensis, SMS), Munich, Germany; 4 TCM practice Hamburg, Mamma Center Hamburg, Jerusalem Hospital, Hamburg, Germany; 5 TCM gynecological practice Berlin, Berlin, Germany; 6 University of Maryland School of Medicine, Center for Integrative Medicine, Baltimore, MD, USA; 7 TCM practice, Berlin, Germany; 8 Competitive Sports Center and College Berlin, Berlin, Germany; 9 Department of Internal and Integrative Medicine, Kliniken Essen-Mitte, Essen, Germany; 10 Sophie-Charlotte High School, Berlin, Germany; 11 University of Traditional Chinese Medicine, Shanghai, China; 12 School of Acupuncture-Moxibustion and Tuina, Beijing University of Chinese Medicine, Beijing, China

**Keywords:** Comparative effectiveness research, Stakeholder engagement, Acupressure, Menstrual pain, Smartphone application

## Abstract

**Background:**

Self-care acupressure might be successful in treating menstrual pain, which is common among young women. There is a need for comparative effectiveness research with stakeholder engagement in all phases seeking to address the needs of decision-makers. Our aim was to design a study on the effectiveness of additional self-care acupressure for menstrual pain comparing usual care alone using different methods of stakeholder engagement.

**Methods:**

The study was designed using multiple mixed methods for stakeholder engagement. Based on the results of a survey and focus group discussion, a stakeholder advisory group developed the study design.

**Results:**

Stakeholder engagement resulted in a two-arm pragmatic randomized trial. Two hundred and twenty women aged 18 to 25 years with menstrual pain will be included in the study. Outcome measurement will be done using electronic questionnaires provided by a study specific mobile application (*App*). Primary outcome will be the mean pain intensity at the days of pain during the third menstruation after therapy start.

**Conclusion:**

Stakeholder engagement helped to develop a study design that better serves the needs of decision makers, including an *App* as a modern tool for both intervention and data collection in a young target group.

**Trial registration:**

Clinicaltrials.gov identifier http://NCT01582724

## Background

Primary dysmenorrhea with an estimated prevalence of 43% to 90% is one of the most common health problems among women younger than 25 years [1] and one of the main reasons for short-term school [2] or work absence, resulting in a significant economic loss [3].

Non-steroidal anti-inflammatory drugs (NSAIDs) and oral contraceptives are commonly used treatments for menstrual pain, but are associated with relevant side effects [4,5]. Acupuncture is widely used by patients suffering from pain [6,7], and a pragmatic trial came to the conclusion that additional acupuncture was associated with improvements in pain and quality of life when compared to usual care alone in women with dysmenorrhea [8]. A pilot trial from the US comparing acupuncture with sham acupuncture in young girls with menstrual pain identified problems in recruitment and compliance [9]. Contrary to acupuncture, acupressure offers the possibility to do a self-care treatment at home. A systematic review of acupressure for primary dysmenorrhea suggested that acupressure might be successful in the treatment of menstrual pain [10]. However, studies have been small and self-care acupressure has been investigated only in two studies from Taiwan [11] and from Iran [12].

To strengthen the available evidence for clinical decision-making studies in the field of comparative effectiveness research (CER) are needed [7]. In particular, diseases that are common and costly for society warrant investigation by CER [13]. CER is the generation and synthesis of evidence that compares the benefits and harms of alternative methods to prevent, diagnose, treat, and monitor a clinical condition or to improve the delivery of care with the purpose of assisting consumers, clinicians, purchasers, and policy-makers to make informed decisions that will improve healthcare at both the individual and population level [14]. From a methodological point of view, a study can be more on the effectiveness or on the efficacy side. ‘Effectiveness’ is defined as a measure of the extent to which an intervention, when deployed in the field in routine circumstances, does what is intended to do for a specific population, whereas ‘efficacy’ refers to the extent to which a specific intervention is beneficial under ideal conditions [15]. The pragmatic explanatory continuum summary (PRECIS) [16] was developed to provide a graphical tool of 10 design domains, which helps researchers to categorize trials as more effectiveness or efficacy focused. Very often clinical studies are placed somewhere within the efficacy-effectiveness continuum [17].

To address the needs of decision-makers, stakeholder engagement had become a central point of successful CER and high quality evidence in the US [18]. Stakeholders should be involved in all phases of research including designing and implementing studies [18]. Stakeholders are defined as individuals, organizations, or communities that have a direct interest in the process and outcome of a project, research or policy endeavor. Stakeholder engagement is defined as an ‘iterative process of actively soliciting the knowledge, experience, judgment and values of individuals selected to represent a broad range of direct interests in a particular issue, for the dual purpose of creating a shared understanding and making it relevant, transparent and effective’ [19]. In Germany, stakeholder engagement as part of CER is new and not yet included into research policy [20].

The purpose of this article is to provide our practical experience with stakeholder engagement in designing a pragmatic study and to present the subsequent study which investigates the effectiveness of additional self-care acupressure for women with menstrual pain compared to usual care alone.

## Methods

### Stakeholder engagement

We used multiple and mixed methods to involve stakeholders. In November 2011 a focus group discussion and a cross-sectional survey were conducted among female college students aged 16 to 25 years suffering from self-reported menstrual pain. Furthermore, a Stakeholder Advisory Group was implemented to design the study and a written Delphi consensus method with international acupuncture specialists was conducted to define the intervention. The aim of the focus group and the survey was to get an understanding of women’s view about their menstruation, related pain and additional symptoms, and potential treatment options such as acupressure and relaxation techniques. Additionally, women were asked whether they owned a smartphone and would use it for a study intervention. The deans of 24 colleges provided by the Berlin Chamber of Commerce were contacted by phone and informed about this pre-study. Those who were interested in participating received written information about the study and after two colleges agreed recruitment was stopped. Participants in the focus group discussion and the survey obtained informed consent. Both pre-studies were approved by the local ethics committee (Approval number: EA1/223/11).

#### Focus group

Seventy-six female students from a college for body care received written and oral information by a researcher of the study team 1 week before the scheduled focus group took place. The focus group discussion lasted approximately 60 min and was carried out in the conference room of the college. A trained moderator and the study coordinator moderated the group discussion using a semi-structured interview guideline that was developed by an expert team and pretested. The interview was digitally recorded and subsequently transcribed. A qualitative researcher coded and analyzed the focus group interview using content analysis supported by the software MAXQDA 10 [21]. Codes were abstracted into different categories and main topics were developed in the analysis process. The results were finally discussed and enhanced with further researchers of the study group.

#### Survey

Two colleges agreed to participate in the survey. The questionnaire consisted of 30 items and was handed out to 124 women who were present in class at the scheduled survey assessment. Women were asked to complete the questionnaire. Completed questionnaires could be returned immediately or could be sent back by mail in pre-paid envelopes (no costs resulted for the participants). The survey questions were analyzed descriptively; only percentages were reported as valid percentages using IBM SPSS, Version 20 for Macintosh.

#### Delphi consensus and stakeholder advisory group

A written Delphi consensus method with international experts on acupuncture and acupressure from three countries (China, Germany, USA) was conducted to specify the intervention. By the Stakeholder Advisory Group the following requirements for the development of the self-care acupressure were defined: (1) as little time as possible to be spent on the acupressure sessions; (2) points should be easy to find without fully undressing; and (3) number of acupressure points should be limited to not more than three.

Potential stakeholders identified out of stakeholders’ categories in CER [19] were contacted by either telephone or email and asked to participate as a representative in the Stakeholder Advisory Group. A female gynecologist, a 16-year-old woman with dysmenorrhea, a female teacher, two acupuncture experts, and a mind-body medicine expert represented the final Stakeholder Advisory Group. Based on the results of the focus group and the survey, the Stakeholder Advisory Group developed the study design and determined the position in the efficacy-effectiveness continuum using the PRECIS tool [16]. The PRECIS tool consists of 10 design domains and was developed to increase transparency in reporting to which extent a trial is more applicable to a real-world setting (effectiveness) or more ideal and experimental (efficacy).

## Results

### Focus group discussion

Seven women (mean age, 21.0 ± 2.7 years) participated in the focus group.

Interviewees described their symptoms including suffering from pain in lower abdomen, abdominal cramps, back pain, headache, and nausea as well as changes in mood such as irritability, anger, and fatigue. Consequently, these feelings of discomfort caused different limitations in daily activities. Altogether the menstruation topic was afflicted with a lot of shame, for example some participants mentioned hesitance to discuss it with their parents due to feeling ashamed. Three women reported that sick leave was regularly required for 1 or more days. As a result of their mood changes, interviewees experienced lack of understanding from their environment and consequently having arguments with their partners and friends.

The participants stated little to no interest in doing relaxation techniques. Impatience was the main reason for this lack of interest. Those young women could hardly imagine integrating relaxation techniques in their daily life and to spend any time on them.

‘*No, I wouldn’t do things like that, because I can’t sit at home, even at home or somewhere else and relax. Impossible. Something constantly enters my head and I’m always anywhere else. I have to do something. Not possible at all.*’ (Quote from a female student)

In comparison to relaxation techniques, the interest in applying self-care acupressure was high if conducted on the days when the pain was present. However, only few women could imagine a preventive application of self-care acupressure before the onset of menstruation.

The participants could imagine applying self-care acupressure several times a day for a few minutes at home or at school. They envisaged participating in a personal introduction about self-care acupressure and favored inclusion of such techniques in school lessons. As an instruction tool they preferred guidance by an App compared to directives on paper. Instructions should include the application time, localization of the relevant acupressure points, and method of pressure.

### Survey

Ninety-eight women out of 124 (mean age, 19.0 ± 2.7 years) completed the questionnaire. Twelve women chose not to participate in the survey, 11 did not suffering from menstrual pain, and three students did not fit the age range requirements. The main results of the survey are presented in Table 1.

**Table 1:** Answers of the women to questions of the survey

	**Women (%) (*n*** **=98)**
Experience of menstrual pain during every menstruation	66
General complaints^a^	
Pain in the lower abdomen	69.8
Abdominal cramps	60.4
Back pain	52.1
Headache	27.4
Nausea	12.6
Other symptoms	10.4
Menstruation impairs daily activity	46.9
At least 2 days absent from school per year	20.4
Use of medication at menstruation	24.5 (96% of them painkillers)
Of those would like to reduce medication intake	79.2
Worst pain ≥6 on NRS	70 (mean 8.4, SD 2.7)
Pain on NRS, mean (SD)	6.9 (2.7)
Would imagine applying acupressure to reduce menstrual pain	45.8
Of those ‘How long would you aim to applyacupressure at the days of pain, if your menstrual pain could be vastly improved?’ (minutes)	
<10	13.6
10-15	56.8
16-20	13.6
>20	15.9
Of those ‘How often would you aim to apply acupressure on the days with pain?’ (times per day)	
1	25.0
2	38.6
3	18.2
>3	18.2
Of those ‘How often would you aim to apply acupressure on the days without pain, if your menstrual pain could be vastly improved?’ (times per day)	
1	65.1
2	20.9
3	11.6
>3	2.3

^a^More than one possible answer.

NRS, Numeric rating scale; SD, Standard deviation.

### Delphi process

The acupressure protocol was based on three Delphi rounds resulted in three predefined points (SP6, LR3, LI4). Nine acupuncturists participated in the Delphi process with three practitioners from China, five from Germany, and one from the US. The self-care intervention within study will require 1 min of acupressure for each point bilaterally, in total 6 min per session, starting 5 days before menstruation, once up to twice daily and when the pain is present twice up to five times daily. The acupressure groups can use usual care defined as all medical and non-medical treatments (except of Tuina, Shiatsu, and acupuncture because of the use of similar pressure points).

### Stakeholder advisory group

**Figure 1:**
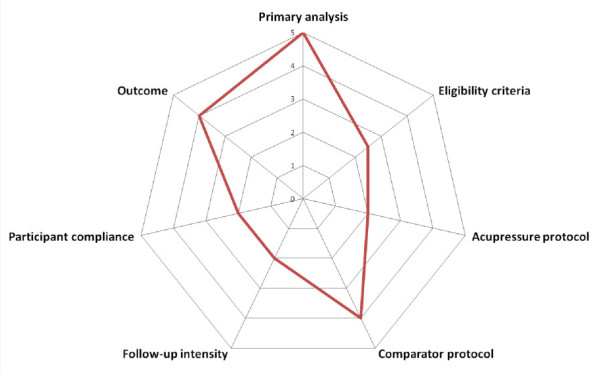
Pragmatic-explanatory continuum indicator summary (PRECIS) for the main study displaying the location of seven study design criteria to be more on the effectiveness side (outer circles), more of the efficacy side (inner circles), or in the middle of the continuum.

A total of three stakeholder meetings took place, complemented by email discussions. The first meeting was conducted before initiation, the second after completion of the survey and the focus group discussion, and the third to finalize the study protocol and to determine the study scores in the efficacy-effectiveness continuum with the PRECIS. The place of our trial in the effectiveness-efficacy continuum was defined more on the effectiveness side for the criteria primary analysis, control protocol and outcome measurements, whereas participants compliance, follow-up intensity, and acupressure protocol were more on the efficacy side, and eligibility criteria was in the middle of the continuum (Figure 1). From the 10 PRECIS criteria practitioner expertise and adherence were not applicable.

Based on the focus group, the survey, and the different expertise in the stakeholder group, the Stakeholder Advisory Group made the following main decisions:

1) It was decided to perform a two-armed study with acupressure as single intervention forgoing the plan to include relaxation techniques as further intervention. Applying relaxation techniques was not considered feasible for young women with dysmenorrhea because the motivation to exercise regularly was low, even when asymptomatic, and relaxation conducted only on days of pain was considered to be ineffective.

2) Only women between 18 and 25 years will be recruited. Women <18 years who were interested in participating in the focus group discussion failed to participate because of missing parental informed consent. Also, the informed consent procedure for under-age study participants becomes much more complex which could result in reduced feasibility for recruitment. However, the prevalence of menstrual pain was high in women aged ≥18 years in the survey and the study hypotheses can be investigated without including under-age women. Furthermore, our experience from the focus group discussion was that asking for parental consent to participate in a study about menstrual pain might cause embarrassment.

3) The intervention will be developed to meet the following criteria: easy and fast to apply with a limited number of acupressure points. The amount of time to spend on self-care acupressure should not exceed 7 min per session.

4) Outcome measures will be chosen according to patient relevance and require only a short time to fill out questionnaires and diaries.

5) The study will include a smartphone application that allows data collection (except baseline) from pre-programmed questionnaires and instruction guidance of the intervention.

### Design of the main study

The study will be a two-armed, randomized pragmatic trial investigating the effectiveness of additional self-care acupressure for women with menstrual pain compared to usual care alone. Intervention duration will be six menstruation cycles per participant, with the primary endpoint after three menstruation cycles and a further follow-up after six menstruation cycles.

A smartphone application (*App*) will be developed with the purpose of collecting follow-up data and to provide educational guidance on self-care acupressure. The App includes a menstruation cycle calculator, notification features, diaries, and questionnaire facilities.

#### Participants

Potential participants will be informed with brochures and posters at universities, colleges, gyms, and doctors’ offices. The study will include women aged 18 to 25 years with dysmenorrhea (defined as cramping pain during every menstrual cycle). Pain has to be moderate or severe and defined as a score ≥6 on the Numeric Rating Scale (NRS) for the worst pain intensity during the last menstruation. Furthermore, to be included in the study women need to have had their menstruation in the last 6 weeks, a duration of menstruation cycles between 3 and 6 weeks, and no prior history of gynecological disease that could be a reason for dysmenorrhea. Additionally, having a smartphone (iPhone or Android) is required.

Women will be excluded if they are receiving or conducting acupressure, acupuncture, Shiatsu-or/and Tuina massage at the moment or are planning to do so in the next 8 months and if they are pregnant or are planning to become pregnant in the next 8 months.

#### Randomization

If a woman meets all inclusion and no exclusion criteria she will be randomized to either the control (usual care only) or the intervention (usual care plus acupressure) groups by simple randomization with an allocation ratio of 1:1, that is 110:110 participants. The randomization list will be included in a safe Microsoft Access database to ensure that it is not accessible during the randomization process of individual participants. The randomization list will be created in SAS (Version 9.2, SAS Inc., Cary, NC, USA) by a statistician. Randomization process will be conducted by the study office at the Institute for Social Medicine, Epidemiology, and Health Economics. To ensure allocation concealment upon inclusion into the study the stuff enters participants into the database and receives the allocation to intervention or control group.

#### Acupressure intervention

A personal training of self-care acupressure will be provided to all participants in the acupressure group. Additionally, information about acupressure (video, pictures, text) will be provided within the smartphone application.

#### Control group

Women in the control group will continue using usual care defined as all medical and non-medical treatment (exceptions are Tuina, Shiatsu, and acupuncture).

#### Outcomes measurement

Primary outcome measure is the mean pain intensity on a NRS (0 = no pain, 10 = worst possible pain) at the days of pain during the third menstruation after therapy start [22]. Participants will be informed that in the event they do not have any pain during their menstruation they have to choose the score ‘0’ at the NRS.

Secondary outcome endpoints are: worst pain intensity (NRS), duration of pain, responder rate defined as 50% mean pain reduction on the NRS on the days of pain compared to baseline, sick leave days, intake of medication against menstrual pain and body-efficacy expectation. Measurements will be done at baseline, during and after the first, second, third, and sixth menstruation. Additionally, credibility of the intervention will be measured after the third menstruation in the intervention group. We will also collect data about expectation, self-reported general change of menstrual pain, adverse events, and adverse effects of the acupressure (only in the intervention group).

#### Data collection

At baseline data will be collected using paper and pencil. For the follow-up data collection a smartphone application (the study *App*) will be developed to run on iPhone and Android operating systems.

This study App will feature electronic diaries for the days of menstruation (8 days maximum) in which study participants will log daily data on their medication intake, type of complaints, and on compliance (duration and frequency of acupressure) if required. Furthermore, it consists of a questionnaire that will appear after the end of menstruation. All data measurements and time points are listed in Table 2.

**Table 2:** Data collection

	**Baseline**	**Cycle 1**	**Cycle 2**	**Cycle 3**	**Cycle 6**
Sociodemographics (age, migration background, education)	x				
Mean pain intensity on the days with pain (NRS)	x	x	x	x	x
Worst pain intensity (NRS)	x	x	x	x	x
Duration of pain (days)	x	x	x	x	x
Sick leave days	x	x	x	x	x
Intake of medication against menstrual pain	x	x	x	x	x
Adverse events and adverse effects^a^		x	x	x	x
Application of other therapies	x	x	x	x	x
Body efficacy expectation	x	x	x	x	x
Credibility of the intervention^a^				x	
Expectation	x				
Type of menstrual complaints^b^	x	x	x	x	x
Compliance^a,b^		x	x	x	x

^a^Only in the intervention group.

^b^Assessed by the diary.

#### Sample size

A sample size of 86 women per group will have a power of 90% to detect a mean difference of 1.5 points on the NRS (primary outcome) using a two-sided *t*-test with significance level of 0.05. Based on effect size in previous acupuncture studies, we assume a mean of 5.5 in the control group and 4.0 in the intervention group, with a common standard deviation of 3 points. We will include 110 women per group (220 in total) to compensate drop-outs. Sample size calculation was completed with nQuery Advisor 6.02.

#### Data analysis

The primary analysis of the primary outcome will be calculated using an analysis of covariance (ANCOVA), adjusted for baseline NRS value (fixed covariate) to test the null hypothesis of equal means in ‘mean pain intensity at the days of pain during the third menstruation’ between the two treatment groups. The analysis will use the full analysis set (FAS, all available data without imputation of missing values) with a two-sided significance level of 0.05. Results will be reported as adjusted group means with 95% confidence intervals and the *P* value for the group comparison. All further analyses on the primary and all secondary endpoints will be considered explorative and no error adjustment for multiple testing will be done. The secondary outcomes will be analyzed for the FAS similarly to the primary analysis, that is ANCOVA, logistic regression, or Poisson regression (depending on the scale and distribution of the data), adjusted for the respective baseline value (when available). The primary analysis of the primary outcome will be repeated based on the per-protocol population. In addition, missing data on the primary outcome will be imputed by multiple imputation techniques. In case of relevant differences in baseline variables between the treatment groups, the analysis of the primary outcome will be repeated with the additional adjustment for these variables. As supportive analysis, mixed model for repeated measures (MMRM) will be fit to compare the two treatment groups with respect to changes in the primary outcome over time. The model will include terms for treatment and time as fixed main effects, an interaction term for treatment by time, and the subject as a random effect. Further details will be described in the statistical analysis plan. The following subgroups will be considered: migration background (yes/no), age groups, anticontraceptive use (yes/no). A detailed statistical analysis plan (SAP) will be developed prior to any data analyses. Data analysis will be performed in SAS Version 9.2 or higher (SAS Inc., Cary, NC, USA).

#### Ethics

The protocol of the main study was approved by the local ethics review boards (Approval number: EA 1/027/12) and the study itself will be conducted according to common standard guidelines (Declaration of Helsinki, Good Epidemiological Practice, WMA General Assembly, Somerset West, Republic of South Africa, 1996). All study participants will provide written informed consent.

## Discussion

To the best of our knowledge this will be the first study that investigates the effectiveness of additional self-care acupressure against menstrual pain in a Western country. Furthermore we used broad stakeholder engagement [19] to plan a study that is useful for clinical decision-making. Menstrual pain is a common problem with a high socioeconomic burden and the advantage of self-care acupressure implemented through an App is that it is easy to apply in a real-life context. Consequently a pragmatic trial was designed to assist healthcare decision-makers with high quality evidence.

This paper presents the impact of stakeholder engagement on the development of the study design and the resulting study design. Our approach represents two main advantages: input of international experts to develop the study intervention and broad stakeholder engagement for study protocol development.

Results of the survey and the focus group discussion were at some points conflicting and contradicting, for example the different amounts of time women are willing to spend for acupressure. Therefore, the Stakeholder Advisory Group was a key element in the decision making process. Its five main decisions had a significant impact on the study design, resulting in the cancellation of a whole previously planned study arm. All phases of the process were fair and transparent to promote a shared understanding among the various stakeholders. As the number of stakeholders’ increase and the perspectives of the group become more diverse, maintaining such transparency could prove more complicated. It is clear that stakeholder engagement requires investment of people, time, and monetary resources. In the study design presented here the process of stakeholder engagement took 8 months and several researchers were involved.

We observed that the desires of the stakeholders and the consensus of experts about the intervention protocol could be opposing. Whereas the interviewees of the focus group could hardly imagine applying preventive acupressure, the experts found it absolutely necessary to achieve a good treatment benefit. We tried to balance the different perspectives of stakeholders with multiple techniques, but the results of stakeholder engagement might have been different if other stakeholders had participated or we had conducted the survey and focus group discussion at different colleges. Another limitation was that we were not able to include a representative from a health insurance company to participate in the stakeholder advisory group. Furthermore, CER is a new concept in Germany and stakeholders are not accustomed to be invited to support research projects and are not aware of the benefit of their participation. Increased public awareness of a ‘help us to help you’ mentality and an importance placed on stakeholders as decision-makers [23] can increase the enthusiasm and likelihood of engagement in the research process.

When applying the PRECIS criteria to our trial we were aware that most design elements were rated to be in the middle of the effectiveness-efficacy continuum. Whereas patient-centered outcomes and a flexible comparator protocol are more on the effectiveness side of the continuum, monitoring of compliance and treatment protocol are more on the efficacy side. Eligibility criteria are moderately pragmatic with inclusion of a limited range of affected women. Using an App with reminders and compliance measures can be seen as the main reason that our trial score was more in the middle of the efficacy-effectiveness continuum. The PRECIS rating was found to be useful in making the elements of the trial design and its difference from a usual care setting more transparent. We rated the trial in a consensus meeting within the members of the Stakeholder Advisory Group. Had the rating process be carried out independently by the individual stakeholders as described in previous studies [24,25] we may have seen different results. However, when non-scientific stakeholders are included in the rating process our approach, which prioritizes group discussion and consensus, might be more efficient in addressing any questions as they arise.

## Conclusion

Our experience with stakeholder engagement as part of CER was beneficial helping us to develop a study design that meets more of the needs of decision-makers. This includes using an App as a modern tool for both the implementation of the intervention and data collection in a young target group. The subsequent pragmatic randomized trial will investigate the effectiveness of additional self-care acupressure for women with menstrual pain compared to usual care alone. The results will strengthen the evidence on self-care acupressure for menstrual pain.

## Abbreviations

ANCOVA: Analysis of covariance; CER: Comparative effectiveness research; NRS: Numeric rating scale; NSAIDs: Non-steroidal anti-inflammatory drugs; PRECIS: Pragmatic explanatory continuum summary; SAP: Statistical analysis plan

## Competing interest

The authors declare that they have no competing interests.

## Authors’ contributions

Conceived and designed the study: SB, LS, DP, CW. Performed the pre-study: SB, LS, CW. Quantitative data analysis: LS, SB. Qualitative data analysis: NM. Stakeholder advisory group: WH, DP, BB, KK, SM, AP, CPN. Acupuncture Delphi consensus process: WH, BB, JH, BK, KK, LL, FL, AM, HW, JZ. Statistics: SR, CW. Wrote the first draft of the manuscript: SB. All authors read and approved the final manuscript.
